# Patient and healthcare provider experiences of hepatitis C treatment with direct-acting antivirals in Rwanda: a qualitative exploration of barriers and facilitators

**DOI:** 10.1186/s12889-020-09000-0

**Published:** 2020-06-16

**Authors:** Janvier Serumondo, Michael J. Penkunas, Julienne Niyikora, Alida Ngwije, Athanase Kiromera, Emmanuel Musabeyezu, Justine Umutesi, Sabine Umuraza, Gentille Musengimana, Sabin Nsanzimana

**Affiliations:** 1 Rwanda Biomedical Center, Kigali, Rwanda; 2 Clinton Health Access Initiative (CHAI), Kigali, Rwanda; 3 University of Maryland, Kigali, Rwanda; 4 King Faisal Hospital, Kigali, Rwanda

**Keywords:** Hepatitis C, Direct-acting antivirals, Qualitative, Sub-Saharan Africa, Rwanda

## Abstract

**Background:**

Direct-acting antivirals (DAAs) are increasingly accessible to patients with hepatitis C (HCV) worldwide and are being introduced through national health systems in sub-Saharan Africa. DAAs are highly efficacious when tested in controlled trials, yet patients treated outside of study settings often encounter challenges in completing the full treatment and follow-up sequence. Little information is available on the influences of successful DAA implementation in sub-Saharan Africa. This qualitative study explored the individual- and system-level barriers and enablers of DAA treatment in Rwanda between March 2015 and November 2017.

**Methods:**

Face-to-face interviews were conducted with 39 patients who initiated care at one of four referral hospitals initially offering DAAs. Ten healthcare providers who managed HCV treatment participated in face-to-face interviews to examine system-level barriers and facilitators. Interview data were analyzed using a general inductive approach in alignment with the a priori objective of identifying barriers and facilitators of HCV care.

**Results:**

Barriers to successful treatment included patients’ lack of knowledge surrounding HCV and its treatment; financial burdens associated with paying for medication, laboratory testing, and transportation; the cumbersome nature of the care pathway; the relative inaccessibility of diagnostics technology; and heavy workloads of healthcare providers accompanied by a need for additional HCV-specific training. Patients and healthcare providers were highly aligned on individual- and system-level barriers to care. The positive patient-provider relationship, strong support from community and family members, lack of stigma, and mild side effect profile of DAAs all positively influenced patients’ engagement in treatment.

**Conclusions:**

Several interrelated factors acted as barriers and facilitators to DAA treatment in Rwanda. Patients’ and healthcare providers’ perceptions were in agreement, suggesting that the impeding and enabling factors were well understood by both groups. These results can be used to enact evidence-informed interventions to help maximize the impact of DAAs as Rwanda moves towards HCV elimination.

## Background

When left untreated, chronic hepatitis C virus (HCV) infection can lead to a suite of negative health outcomes, including cirrhosis, hepatocellular carcinoma [1–3], and reduced quality of life [4]. Yet patients who achieve a sustained virological response (SVR) experience all-cause mortality at a rate that is nearly indistinguishable from the general population [5]. Early HCV treatments utilized interferon-based drugs that required up to 12 months of injections and resulted in SVR for approximately 50% of patients [6, 7]. These patients encountered a range of physiological and psychological side effects [8, 9]. A new generation of drugs—direct-acting antivirals (DAAs)—hold the promise to revolutionize HCV treatment. DAAs have demonstrated an efficacy up to 95% in clinical trials, are administered orally rather than subcutaneously, and display highly tolerable side effect profiles [10]. If treatment is adhered to, DAAs have the potential to save the lives of many of the estimated 71 million individuals with chronic HCV worldwide [11].

DAAs were first introduced in Rwanda in November 2015 and have since replaced less-effective interferon-based therapies [12]. Patients with HCV and the Rwandan health system have been anticipating the establishment of DAAs as standard care. Many of the first cohort of patients, some of whom likely delayed care until the DAAs became available [13], have now completed treatment. Nearly 92% of patients who completed the full course of treatment achieved SVR [14], an overall result similar to that found in real-world investigations of DAAs carried out in high-income settings [15–22]. However, in Rwanda, three out of ten DAA patients did not complete treatment or were missing post-treatment HCV RNA data in the national HCV database. This large amount of incomplete data means that some patients who experienced a virological failure are likely unaware of their status and remain at risk for decompensation and act as a reservoir for continued spread of the virus [16]. These patients could have encountered challenges as they navigated the HCV cascade of care [23, 24] and/or persistent documentation gaps may be influencing data completeness.

Qualitative investigations into patients’ experiences receiving care and the practices of healthcare professionals providing HCV treatment can yield valuable information on access barriers and potential solutions for overcoming these obstacles [25]. For example, Jordan and colleagues [26] described patients’ suspicion towards healthcare providers and shortcomings in linkage as obstacles to engaging in care. Additionally, US- and UK-based studies employing qualitative methods highlighted the fact that patients with HCV commonly do not experience noticeable physical symptoms [27, 28] and this can act as an impediment to seeking treatment [29]. Co-occurring physical and psychological ailments have also been cited as potential barriers to care as patients consider engaging in HCV treatment [30]. Conversely, qualitative studies have reported that positive relationships and open communication with healthcare providers are factors that promote engagement and adherence to treatment [31, 32].

Gaining a thorough understanding of the individual- and system-level barriers and enablers of HCV treatment can help the Rwandan healthcare system maximize the positive impact of DAAs. A close examination of HCV care and treatment from the perspectives of patients and healthcare providers will illustrate the reasons why some patients did not remain engaged in care and could reveal issues related to clinical documentation. As such, the primary goal of this study is to explore patients’ and healthcare providers’ perceptions of barriers to and facilitators of DAA initiation and completion.

## Methods

### Recruitment and consent

The national-level database containing information for patients who initiated DAA-based treatment between November 2015 and March 2017 was used to create a sample of potential participants. Patients treated at the four referral hospitals in Rwanda (King Faisal Hospital, Rwanda Military Hospital, University Teaching Hospital of Butare, University Teaching Hospital of Kigali) during the time period of interest were categorized by treatment outcome: achieved SVR, completed treatment but experienced virological failure, or non-virological failure (lost to follow-up or missing HCV RNA data necessary to determine SVR). Patients within each treatment outcome classification were assigned a randomly generated number in Microsoft Excel, stratified by hospital. We aimed to recruit a sample of 16 patients from each treatment outcome category, four from each referral hospital.

Recruitment phone calls were made by a member of the Viral Hepatitis Unit within the Rwanda Biomedical Center (RBC) following the randomly generated numbers sequentially by hospital. Inclusion criteria were intentionally broad; all patients initiating DAA-based therapy between November 2015 and March 2017 were deemed eligible as long as they were at least 18 years old and available for a face-to-face interview. During the initial telephone call, each potential participant was read a recruitment script that provided a brief overview of the study and was asked to participate in a face-to-face interview. If the potential participant agreed to be interviewed, a meeting time and location was set, with the understanding that the interviewer would travel to a location convenient for the participant. Contact was made with 46 patients. Three individuals declined to be interviewed, and six individuals agreed to be interviewed but were not available during the data collection time period. Of the 39 patients who participated in an interview, 15 achieved SVR, 10 experienced virological failure, and 14 were classified as non-virological failure.

Central-level administrative records maintained by RBC were used to identify healthcare providers who managed the care of patients with HCV during the time period of interest. As with the recruitment of patients, a member of the Viral Hepatitis Unit at RBC called potential participants to describe the project and asked if the provider was interested in participating in the study. Three of the four physicians who provided specialized HCV care during the study period agreed to be interviewed. Seven nurses were identified as providing HCV care during the time period of interest and all agreed to participate in the interviews, bringing the number of healthcare providers included in this study to ten. All patients and healthcare providers provided written informed consent prior to the interview. Participants were compensated 5000 Rwandan Francs (approximately $5.75) for their time.

### Interview methods

Data collectors underwent a hands-on training on qualitative methods specific to this study, which included didactic presentations and role-play exercises with critique. Semi-structured, face-to-face interviews were conducted in a private area at a place in the community convenient for the participant, such as a health facility, their home, or their place of work. The interview guides for patients and healthcare providers were developed for this study specifically and are presented in the supplementary materials. Questions for patients focused on their experiences receiving care, perceived facilitators and barriers of treatment, and their recommendations for improving HCV care in Rwanda. Healthcare providers were interviewed in a private area of the hospital where they worked. Questions for healthcare providers explored their experiences in providing HCV treatment, challenges they and their patients encountered while providing and receiving care, and their opinions on how to strengthen HCV care and treatment processes. The semi-structured nature of the interviews allowed both patients and healthcare providers to steer the discussion in directions that were meaningful to them, even if the topics were outside of those presented on the interview guides. All interviews were audio-recorded using an encrypted, password-protected digital audio recording device. Audio files were transcribed verbatim in Kinyarwanda and translated into English by a third-party market research company in Kigali, Rwanda. All audio and text files were transferred and stored on encrypted digital devices.

### Data analysis

Analysis was guided by a general inductive approach, a methodology that allows for more rapid analysis of qualitative data compared to others (e.g., grounded theory), yet can result in robust findings when applied rigorously [33]. A general inductive approach was chosen as the most appropriate analytical methodology here due to our a priori objective of identifying barriers to and facilitators of HCV care; we did not aim to test a specific hypothesis or validate a theoretical model.

Two coders with backgrounds in qualitative health research began by closely reading the entire library of transcripts from the healthcare provider interviews to develop an overall understanding of the themes and events presented. Coders approached the raw data from the stance of identifying the core meaning of the text as it related to the primary evaluation objective. An overall coding scheme was constructed with upper-level (i.e., more general) categories defined by the aims of the evaluation: identifying key barriers and facilitators of successful treatment. More specific, lower-level codes were created from the statements presented in the raw text. Transcripts for interviews conducted with patients were received on a rolling basis and were coded in line with the developed codebook. The two coders met regularly to discuss the overarching themes emerging from the healthcare provider and patient interviews and to refine the coding system as necessary. Discrepancies were resolved through conversation with direct consideration of the original data. Analysis was conducted using Dedoose V8.0.42 [34].

## Results

Interviews ranged from 13 to 78 min in length, with the majority of interviews lasting between 30 and 45 min. Tables 1 and 2 display the characteristics of participating patients and healthcare providers, respectively.

**Table 1:** Characteristics of patients participating in the qualitative interviews, by treatment outcome

Patient characteristics	SVR	Virological Failure	Non-Virological failure	Total N
**Sex**				
Female	11	3	8	**22**
Male	4	7	6	**17**
**Age Category**				
Under 45	2	–	2	**4**
Between 45 and 65	5	7	7	**19**
Over 65	8	3	5	**16**
**Place of Residence**				
Kigali	5	4	6	**15**
Outside Kigali	10	6	8	**24**
**Education Level**				
None/Not Available	–	–	2	**2**
Primary	6	3	4	**13**
Secondary	5	5	3	**13**
University	4	2	5	**11**
**Occupation**				
Farmer	2	2	4	**8**
Professional	–	1	1	**2**
Public Servant	2	1	1	**4**
Religious Worker	1	–	–	**1**
Retired	4	2	4	**10**
Self-employed	2	3	1	**6**
Unemployed	4	1	3	**8**
**Hospital**				
University Teaching Hospital of Butare	4	2	4	**10**
University teaching Hospital of Kigali	4	4	4	**12**
King Faisal Hospital	4	2	3	**9**
Rwanda Military Hospital	3	2	3	**8**
**Insurance Type**				
Public	10	6	6	**22**
Private	2	1	3	**6**
None	3	3	5	**11**

**Table 2:** Characteristics of healthcare providers participating in the qualitative interviews

	Total N
**Sex**	
Female	6
Male	4
**Experience as medical professional**	
Less than 20 years	6
20 or more years	4
**Experience providing HCV care**	
Less than 5 years	5
5 to 10 years	3
More than 10 years	2

### Barriers – patient perspective

#### Lack of knowledge around liver health, HCV infection, and DAA treatment

Patients described a need for additional information about the disease, its symptomology, and mode of transmission. In other cases, patients made statements that illustrated how they could benefit from education regarding liver health, HCV transmission, and HCV clinical presentation. This first quotation demonstrates patients’ misunderstanding of how one can become infected with HCV.

*Is [HCV] heritable? … What is it? It means if there is someone in a family who had suffered from it before and it could be inherited … that is what I thought, then … I asked myself whether one of my parents could have had it because I don’t have my parents.*

SVR achieved, 45 years old

As illustrated by the excerpt below, patients often had little knowledge of the liver and signs or symptoms related to HCV infection.

Patient: *There came a time though when I had chest pain and I became interested in finding out the symptoms of hepatitis, and the people I asked could not give me a good answer. So I lived with uncertainty.*

Interviewer: *Who did you ask?*

Patient: *The people who I live with...I said, “I’m just getting chest pain, is this where the liver is?” They couldn’t answer...*

Interviewer: *Have you found an answer to your query regarding where the liver is located?*

Patient: *No, not yet.*

SVR achieved, 70 years old

Despite having received DAA therapy, patients lacked a clear understanding of the monitoring requirements and assessments needed to determine their treatment outcome. Some patients were unaware that they were to wait three months after completing the treatment in order to determine SVR, as shown in the following quotation:

*Before … I completed [the tablets] and rushed to go back there and she told me, when I met the doctor she told me: “Come back after three months*”.

SVR achieved, 45 years old

Similarly, the following patient did not return to their physician with the result from the final HCV RNA test meaning this physician was likely unaware of the patient’s outcome and the test results were not recorded in the patient’s clinical file.

*[The doctor] told me that “if you finished the medicine the next thing is to go back to [the laboratory] and get the medical tests and see if the viruses in the blood are gone.” That is when I went back to [the laboratory] and I got the medical tests done like it’s normally done and they gave me the results and told me that … it was written “none detectable” and I stopped going.*

Non-virological failure, 45 years old

The following quotation illustrates clearly that some patients were cognizant of their need for additional information and that supplementing DAA therapy with education could be beneficial to patients.

*Patients go there being like … like given up hope. Therefore, then that person; I can say a counselor can go through [the process with] them and talk to them … that thing can be very good.*

Non-virological failure, 58 years old

These discussions illustrate the need for additional HCV and treatment-related information. Physicians have little time to counsel patients on topics of liver health and the specifics of HCV, and the development of targeted educational materials could be complemented by formal informational sessions hosted by peers or other healthcare providers supporting patients with HCV.

#### Difficulty in accessing treatment and testing due to financial hardship

Among the barriers encountered by patients, the costs associated with treatment were noted frequently. The DAAs provided during the study period were, at most, only partially covered by insurance companies. Many patients had to pay out-of-pocket for the DAAs and the associated HCV RNA tests (performed prior to treatment and at post-treatment week 12). Financial hardship often determined whether patients completed the full treatment regimen or underwent HCV RNA testing. Treatment was seen as a burden to themselves and their families.

Interviewer: *It means how many times did you take the medications?*

Patient: *There are two which I was able to pay.*

Interviewer*: There are two which you were able to pay. You took, you took them two times and it was completed?*

Patient: *No, it was supposed to be that of three months but … .that of [the third] month I didn’t find them.*

Interviewer: *You went there twice only. Is there any other [test] which you didn’t take?*

Patient: *Yes, the money was much and I wasn’t able to get them.*

Non-virological failure, 49 years old

Patients who were able to complete treatment often had to go to great lengths to acquire the financial resources necessary to do so. The quotation below illustrates how individuals had to liquidate household assets to finance treatment.

*It was difficult to me. It was difficult because finding all that money every month was not an easy thing. It means that sometimes I had to sell other stuff from the household … I mean I have made the household poor...I sold the trees of my forests*.

Non-virological failure, 58 years old

In addition to the DAAs and HCV RNA testing, many patients also mentioned having to pay for transportation to the referral hospital, the pharmacy, and laboratory testing site. These trips were often financed through borrowing money from relatives, and appointment cancelations were a source of frustration for patients who relied on others financially.

*The suggestion … is that I was from far away … and really I don’t even know where to stay [in Kigali] and the financial means were limited … what I had been asking … because I had been asking from relatives like those whom I [helped] when I still had strength; then some started to switch off their telephones. Then, when [at the hospital] they do not do … they tell me that [healthcare provider] can’t give me a service … that … when they don’t respect the appointment they gave me, they are making my plans fail … you too can understand if someone comes on transportation s/he got from others and be told to go back; you see that’s a problem*.

Virological failure, 71 years old

A large amount of resources were necessary for completing the entire treatment package, from diagnosis and DAA prescription to medication pick-up and HCV RNA testing. Sourcing money from relatives or selling assets was burdensome to patients and acquiring the money necessary to pay for HCV services was a barrier to remaining engaged in care. Some patients found themselves unable to either pay for the medications or the HCV RNA testing, leading them to discontinue treatment. Access to financial means was likely also a deciding factor for other patients to not take up DAA treatment after being diagnosed with HCV.

#### Cumbersome treatment process and unpredictable appointment schedules

Experiences in care were frequently described as challenging and requiring a large degree of effort from the patient. Following the full HCV cascade of care was complicated by the fact that service sites were geographically disconnected from one another and required patients to travel between the referral hospital, pharmaceutical distribution hub, and specialized laboratory. Additionally, the appointments patients scheduled with their healthcare providers were sometimes not honored, requiring repeated visits to the referral hospitals.

*Another time I can say which was hard for me, is when I got done biopsy. It’s to take a small sample of the liver and put … and put in a thing and they gave me to take it to [hospital] to get it tested by myself. As I asked myself that, “I see it is difficult to … to take this small sample from the liver and it requires to bring it by myself to another hospital, to test it, and they will give you results and bring them back”. I wasn’t pleased by that service because the … I went scared.*

Virological failure, 50 years old

Some patients reported challenges in having their previously scheduled appointments honored upon arrival at the health facility. Some patients were requested to reschedule their appointment on another day while others reported waiting for long periods to see their healthcare provider.

*Then, what I can recommend them, is to receive a patient in the appropriate time when s/he comes to them … when s/he comes to them; without, “Go and come back later, go and come back later” or without a patient sitting there from morning … till the evening.*

SVR achieved, 58 years old

The patient quoted below articulates his desire to lessen the burden presented by the cumbersome treatment procedures by bringing DAAs closer to where patients live.

*Those medicines, they should put them closer to where they are, not saying that a citizen from [outside of Kigali] must go to take them from Kigali, they will put medicines near to people, uhm, like at health center or hospitals near citizens.*

SVR achieved, 76 years old

Participating patients often stated that the full treatment cascade was difficult to follow and required them to travel to several health facilities. Patients needed to visit different facilities to meet with their physician, fill their DAA prescription, and undergo HCV RNA testing. While being required to travel frequently between facilities, patients also encountered barriers in accessing services because of canceled or postponed appointments. Patients who traveled from rural areas to the city of Kigali, where nearly all of the HCV services were located, were likely demotivated after having their appointments rescheduled, especially if this negatively impacted the timing of other services they had scheduled or had implications for how they would travel back to their homes.

### Barriers – healthcare provider perspective

This section describes barriers encountered from the perspectives of physicians and nurses who managed the care of patients with HCV. These interviews were intended to complement those conducted with patients by identifying barriers present at the facility- and system-level.

#### Heavy workloads and lack of HCV training

Doctors and nurses reported heavy workloads, particularly those who managed the care of patients with HIV in addition to patients with HCV. The ratio of patients to providers is high, leading to some difficulties in scheduling appointments. Only four physicians were authorized to prescribe DAAs during the time period under study. These physicians were supported by a small cadre of nurses, but nurses reported a need for additional HCV-specific training.

Provider: *So when I have a lot of patients for hepatitis, others for HIV, I give appointments to some of them by saying, "Come tomorrow so that I can take care of you like that so that I can be able to "...and when I ask them they agree, they can also see that I am alone and they, they facilitate me.*

10 years’ experience

Nurses, in particular, requested additional training on how to appropriately manage patients with HCV. Doctors and nurses also mentioned being unaware of when treatment guidelines are updated and how to follow the new procedures.

Provider: *Let’s say, like in other programs like HIV and TB, for those you can see that they are reinforced. For those, trainings take place and you find a person in charge of [a disease] here in the institution. A person in charge of HIV and who follows up with what is related to it, who attends all those trainings, and who knows those patients who have a problem. S/he is the one who distributes medicines...but hepatitis there is none.*

12 years’ experience

From the perspectives of healthcare providers, their heavy workload and need for additional training were highlighted as barriers to successful treatment for patients. Physicians’ time was in short supply, which likely necessitated the rescheduling of appointments, as was mentioned by patients. Nurses were clear on their need for additional HCV-specific training similar to that conducted for nurses caring for patients with other infectious diseases.

#### High cost associated with HCV treatment

Healthcare providers recognized that patients’ limited financial resources could lead to challenges in either engaging in or completing treatment. Patients’ inability to pay for DAAs, HCV RNA testing, and transportation were all mentioned by healthcare providers as barriers. This first quotation exemplifies the difficulties providers witness as patients attempt to fund their DAA-based therapy.

Interviewer: *What are the main challenges you come across within providing care to the patient of hepatitis C?*

*Provider: The main challenge is the challenge of means. Patients are required to have medical tests but there you find they do not have means. The second challenge is medication; medications were expensive and were not affordable to all.*

13 years’ experience

Beyond paying for treatment, patients also had to pay for HCV RNA testing services, which constituted an additional financial burden.

*The one who could not be able to find money for the viral test would go back [home], whether he would die or go somewhere else, I don’t know.*

33 years’ experience

Healthcare providers recognized that the centralized nature of HCV services required some patients to travel extensively. The cost associated with transportation was also noted as a barrier to completing treatment.

Provider: … *But the problem that I have is people who did not came to pick up the drugs, while we called them, those whom their viral load results recommend them to take drugs. Some are the ones who have issue of not having the insurance, and they are unable to get medical tests. Others are people who came from [outside of Kigali], who are unable to get [a bus] ticket which brought them here probably. It’s a poverty* issue.

Interviewer: *It’s the poverty issue that you think can … You called them, but they do not yet come to pick up the drugs. Do you think it’s because of the poverty issue?*

Provider: *Right. They have not come yet. There are some who tell us, “For sure I have no [bus] ticket”.*

Nurse, Female, 10 years’ experience

Some decisions made by healthcare providers were influenced by a patient’s ability to pay for the full course of treatment.

*Before 2017, I think that it was still difficult; I think that in 2016, it was still difficult. Because that time the doctor would tell [the patients], “Do you need medicines so that I prescribe them to you? They cost …*” *We told them their price immediately so that s/he understand if s/he will afford it, and if s/he could afford it, then the doctor prescribed them to him/her, if not affordable, told him/her, “If you find means, come back …*”.

8 years’ experience

As described by healthcare providers, patients’ inability to pay for DAAs, HCV RNA testing, and transportation to health facilities were substantial barriers to engaging in and completing care. These conversations with healthcare providers echo those held with patients, and further emphasize the degree to which financial constraints impede HCV care and treatment. To date, changes to the DAA financing scheme are currently underway in Rwanda, but the results here demonstrate that attention must also be paid to peripheral costs in attaining HCV care and treatment, such as those for laboratory services and transportation.

#### Challenges in information availability

Healthcare providers cited limited information availability as a system-level barrier that has downstream impacts on the care of patients. This concept is two-fold. First, healthcare providers state that patients themselves are misinformed about treatment and have received different information from various healthcare providers and are therefore confused about their treatment options. Second, the nurses describe a breakdown in communication between them as front-line providers and the central-level administration.

*I see we don’t have the same understanding on hepatitis, either here in hospitals, either in districts, either in health centers, we have very different information about it, so that a patient may come from maybe the health center being told, “Be aware, maybe don’t do this again, never eat fatty foods again, it is forbidden” And when s/he reach to [the doctor], [the doctor] tells him/her, “No, no, no, you normally get a little milk, drink it.” And you find that s/he is a bit [confused], in fact, they still have problems. They need to be taught, they need … ehh real information, I can say, which is general on problems.*

8 years’ experience

The nurses quoted below suggest the central-level administration could facilitate improvements in communication, information sharing, and policy creation. Solidifying these communication channels would then enable nurses to be better informed care providers.

*What could have been improved as I told you above was firstly to have common national policies. I mean guidelines, [the government], as an institution, which is in charge of implementing the national health system, should link them to those of referral hospitals, and they should work together, not working as two separate institutions. Working on the same thing; that is the thing they can bring together.*

13 years’ experience

*And also communication is much more needed, we need [to know], you to tell us that medications are available, free of charge reagents are available, or reagents for sale are available, so that we are aware of all of that because when you know about it, you have [information] to explain to the patient.*

33 years’ experience

Together, healthcare providers pointed towards communication lapses between different levels of the health system as a barrier to providing high-quality care to patients. Mirroring the perceptions of patients, healthcare providers also stated that patients often do not possess a firm understanding of HCV or its treatment. Providing additional education to patients and improving communication pathways between related health agencies could be beneficial to patients and healthcare providers alike.

#### Difficulty in accessing diagnostic technology and results

Healthcare providers cited the lack of easily accessible diagnostics as a barrier to initiating HCV treatment and as a reason for why patients sometimes do not undergo the final HCV RNA test required to determine SVR. This system-level barrier relates to patients’ experiences having to sometimes travel great distances and pay large sums of money to receive the necessary HCV services.

*We need means for doing [a viral load] test as soon as possible, the need of means to look for patients who can’t know that they are sick, like I said. That is where equipment can be placed, and they are available, which make people be aware that they are sick, they diagnose it as soon as possible, they are put on medicines, and they are followed up, without travelling to the district hospital, that goes to the referrals hospital.*

32 years’ experience

In the following excerpt, a nurse describes a situation where a patient had completed a blood draw, but the HCV RNA results were delayed in being transmitted to the health facility, which subsequently delayed the initiation of treatment.

Provider: *Getting results, the blood samples of patients, taking a lot of time to come, waiting for RBC to bring them in, so when I call them ... we are not ... still ... So you cannot know where you can get the answer. I usually get results too late and I get to the point where I call my patients and find that some of them are dead and that makes me very sad.*

Interviewer: *That is a challenge.*

Provider: *That is a challenge that makes me very sad. If there would be a prompt service, or if we should have [information] so that we can know how the process is. It can be easier for us.*

10 years’ experience

The example quotation from the nurse below illustrates that stock-outs of laboratory commodities also negatively effect the diagnostic process for patients.

*“I found that reagents are over.” That’s what [patients] told me and then s/he goes back home. It means [the reagents] should be available all the time. I think that it can help patients very much, because there are some times when the patient goes in the laboratory and finds that reagents are finished.*

33 years’ experience

Difficulties in accessing diagnostic services due to the centralized nature of the equipment and commodity stock-outs are clear impediments to beginning treatment and determining if SVR has been achieved. Laboratory decentralization efforts are currently underway in Rwanda and the results here illustrate the potential impact of bringing these services closer to patients.

### Facilitators – patient perspective

The following section describes enablers of care from the perspectives of patients. The interviews identified specific contextual features that acted as supports for patients as they progressed through care.

#### Patients’ positive relationships with providers and trust in the health system

The illustrative quotations below show the positive relationships patients shared with the HCV care providers and patients’ gracious attitude towards the government for offering treatment for HCV. These relationships comforted patients who were diagnosed with HCV infection and contributed to their continued engagement in care.

*I can appreciate all the doctors because they could tell we were all scared of this disease. Doctors are geniuses, he could see and tell if you were scared, and he will befriend you and you would talk and tell you everything you want. He would listen to everything you had to say, both what made and didn’t make sense.*

SVR achieved, 43 years old

The quotation below exemplify how grateful patients were to be able to access DAAs and illustrate the positive attitude patients have towards the government for making these drugs accessible to them.

Patient: *It’s our government that encouraged me, my country.*

Interviewer: *How?*

Patient: *You cannot imagine how many people were suffering from the disease and how the government helped to access the drugs; it was years that people were looking for [treatment]. Many people were afraid, some people had died, yes there were [people] who are dead. So, I cannot forget that governments help us a lot, it’s really like Rwandans.*

Virological failure, 46 years old

Patients’ overall sentiment towards HCV healthcare providers was positive, even though patients had also reported frustrations when their appointments were sometimes not kept by providers. The degree of positivity conveyed by patients towards their healthcare providers and the central government likely helped them to remain engaged in care and allowed them to overlook some of the inconveniences they encountered while undergoing treatment.

#### Support from family and community members

In addition to the positive relationships patients shared with their healthcare providers, the support patients received from their family and social network helped them to remain engaged in care. The quotation presented below illustrates the social and financial support patients received from their families, friends, and neighbors while they were undergoing treatment for HCV.

Interviewer: *Then, from when you were sick, your relationship with people you live within your home or even neighbors, relatives and even friends, their attitude toward you, how did they take your illness? Was there a change in their attitude toward you?*

Patient: *No, we live well together.*

Interviewer: *The way they were socializing with you; were there visiting you; how was it?*

Patient: *Uhm [agreeing], they visited me, even all my neighbors.*

Interviewer: *Is there anyone who kept their distance and feel …*

Patient: *[Laugh].*

Interviewer: *Being afraid of you or you, feeling isolated from the public?*

Patient: *No, they visited me. I am in a Christian family and God’s child, they visited me and gave me social and economic support and I really felt happy then.*

SVR achieved, 58 years old

The assistance provided by family and friends of patients consisted of both social and financial support. These supports acted to help patients progress through treatment and facilitated patients in accessing care. The experiences reflected in the quotations above and throughout the patient interviews also illustrate that patients encountered very little or no stigma while undergoing treatment for HCV.

### Facilitators – healthcare provider perspective

From the interviews conducted with healthcare providers, three facilitators of care emerged. The following section describes these enablers.

#### Lack of stigma and a supportive community

When DAAs were first introduced in Rwanda, physicians often scheduled appointments with all of their patients with HCV on the same day, leading to the formation of an informal support group made up of patients who met regularly at the health facility. From the perspective of healthcare providers, the ability for patients to interact with their peers was a source of encouragement for those undergoing treatment for HCV. Additionally, the lack of stigma around HCV and support from the community facilitated patients’ continued engagement in care.

*The fact of being in a group like those first ones we know, it helped them, [because] you can feel you are alone, then feeling you are the only one can cause you to be depressed, to feel that there is nothing you are working for, that those medicines will not heal you and then not be able to … To listen from your colleague maybe to tell you, “For me, I got healed or for me it is like this” so that it gives encouragement to continue.*

12 years’ experience

The lack of stigma around HCV in Rwanda is evident in patients’ reports of being accepted by members of their community after diagnosis and is expressed explicitly by the healthcare provider quoted below.

*It means this fear and having stigma in the society, it didn’t happen. Even their relatives, those who bring them to the health facility, those who take care of them, you couldn’t find them with fear, because this thing, we had first removed it, in those news [stories] we provided on television, on radio.*

32 years’ experience

As was reported by patients, healthcare providers referred to a supportive community of family members and peers as factors that facilitated care for patients with HCV. The positive effect of the informal patient support groups that formed spontaneously suggest that more formalized peer-to-peer counseling could also impact patients’ experience in care, potentially leading to higher treatment completion rates.

#### Dedication of healthcare providers

Participating healthcare providers often mentioned the efforts they put into following-up on patients and educating themselves on HCV care and treatment. This degree of monitoring likely positively impacted adherence to treatment, encouraged continued engagement, and provided an avenue of rapid detection of arising issues.

*You tell the patient that, “Let’s agree, I don’t want you to come and miss me, before coming first call me, this is my phone number. A telephone is not very expensive, they are easy to find.” And s/he comes knowing that s/he will find you, not the other [patient] who will come and wait, having travelled for nothing. To have the support to get all information they need, whenever they need it; and s/he calls you or when s/he knows that you are not [answering the call], s/he sends you SMS and you reply to it later. It’s the greatest support we gave to patients that help them to have adherence.*

32 years’ experience

The nurse quoted below pointed to the success she saw in HCV treatment as a source of motivation for continuing to support patients and seeking out additional HCV-related information.

*What motivated me is how the hepatitis C medicines are available and people are getting cured. I have a database of people whom they found got cured because their first and second medical test resulted negative, and that encourages me to keep taking care of hepatitis C patients through being closer to them, making follow-up day to day on how they feel, checking the reality of what they say on medicine [adherence], checking really if the hepatitis C gets cured. So making that follow-up and through searching and reading the internet made me like this job. I did not attend any related training, but because of being very interested in the work, I read books and read also on the internet, all of those made me more interested.*

10 years’ experience

Providers were motivated to provide high-quality care to patients and the thorough follow-up they provided likely helped form the positive relationships mentioned by patients. Healthcare providers’ dedication as medical professionals also inspired them to seek out additional information on HCV through self-study, which is an illustration of their genuine motivation to support patients.

#### Patients experience only minor side effects and are motivated to engage in care

Healthcare providers also reported that patients experienced only relatively mild side effects while taking DAAs. The ease of taking the medications and a patient’s motivation to remain engaged in care were cited by healthcare providers as facilitators to successful treatment.

*I think the reason is that those medicines do not normally have side effects. If a patient takes medicines for one month and finds he has no problems with those, s/he also takes the second month, the third month, and as result the patient takes medicines adequately.*

14 years’ experience

*Knowing that you have that chance of knowing a person the disease killed, and you are aware that you are going to get well, you are no longer going to die; I can say that those are the two things, two which drive adherence and make Rwandans patients take medicines well, as it is intended. The third one which is visible, when you are going to look others in other countries they have, comparing for example in America, in America, hepatitis C, it is a disease for people who drug themselves using needles, they are young, coming from a society which are bad, which are poor, or have lost their mind like that. Hepatitis C patients in Rwanda, it is a diseases for old people, the average of people we have, they are more than 40 years old, we have those who are between 70 and 80, we give medicines, we have been giving them medicines and get cured, they are old people whose mind is normal, different from, by comparing ourselves with Americans who have people with hepatitis C which is a disease for drug abuse people, they have lost their mind. That is also something which, whether the patient you talk to, they listen, are mature, are responsible, which make them take medicine.*

32 years’ experience

From the perspectives of healthcare providers, the relatively mild side effects associated with DAAs were a substantial facilitator for patients remaining engaged in care. Patients encountered a suite of negative side effects when treated with prior generations of HCV drugs, and the much more tolerable profile of DAAs likely led to patients remaining in treatment who may have otherwise terminated prematurely. Patients’ motivation was likely also influenced by their understanding of the importance of not discontinuing and their sense of personal responsibility in completing the full course of DAAs.

## Discussion

Findings from face-to-face interviews with patients and healthcare providers illustrate that several interrelated factors act as barriers and facilitators that influenced patients’ engagement in HCV care. The primary barriers included challenges in information availability and communication pathways, financial difficulties faced by patients, a centralized care pathway, over-worked healthcare providers, and difficulties in accessing diagnostics. Enablers that lessened the impact of barriers included the positive relationship patients shared with healthcare providers, a lack of stigma around HCV and the subsequent support patients received from their communities, the high degree of dedication among physicians and nurses, and patients’ motivation to remain engaged in care combined with the mild side effects associated with DAAs (Table 3). There was a large degree of overlap in the opinions expressed by patients and healthcare providers, suggesting that the impeding and enabling factors were well understood by both stakeholder groups.

**Table 3:** Summary of barriers and facilitators from the qualitative data

	Barriers	Facilitators
**Patient perspective**	Lack of knowledge around liver health, HCV infection and DAA treatment	Patients’ positive relationships with providers and trust in the health system
	Difficultly in accessing treatment and testing due to financial hardship	Support from family and community members
	Cumbersome treatment process and unpredictable appointment schedules	
**Healthcare provider perspective**	Heavy work-loads and lack of HCV training	Lack of stigma and a supportive community
	High cost associated with HCV treatment	Dedication of healthcare providers
	Challenges in information availability	Patients experience only minor side effects and are motivated to engage in care
	Difficulty in accessing diagnostics technology and results	

Overall, patients possessed low levels of knowledge around HCV symptomology, transmission, and the treatment schedule. This concept was echoed by healthcare providers, which demonstrates the pervasiveness of this barrier. Previous qualitative studies have identified low levels of HCV knowledge as a barrier to care initiation and to remaining engaged in care [35–37]. As described by Swan and colleagues [35], patients’ inability to determine whether a particular health problem was due to HCV has implications for patients’ treatment seeking behavior. Patients in the current study were also misinformed about HCV transmission pathways and symptomology. Of note, patients interviewed here were generally motivated to fill these knowledge gaps. But, possibly because healthcare providers were perceived as being too busy to answer their questions, patients sought information from non-expert sources, potentially perpetuating misunderstandings [37]. These misperceptions can have negative consequences on personal health, such as not seeking care for chest pain, but can also hamper elimination efforts if low HCV knowledge results in continued transmission.

Difficulties in accessing the financial resources necessary to pay for the DAAs, HCV RNA testing, and transportation to and from health facilities also constituted a substantial barrier faced by patients. Participating patients and healthcare providers routinely cited costs as a determinate to whether patients began treatment or remained in care. In their qualitative study exploring barriers to care encountered by HCV patients in the United States’ Veterans Affairs medical system, Rogal and colleagues [38] noted that the cost of treatment hindered patient engagement, although treatments costs in the study location, the United States, were much lower ($5 to $10 per month) than in the current study. DAAs in Rwanda were at most partially covered by participating insurance schemes and would have cost several months of income for a typical Rwandan family. Rwandan patients reported borrowing money from friends and family members or selling off assets to pay for transportation, DAAs, and the ancillary HCV RNA testing. Healthcare providers were acutely aware of the financial burden HCV treatment could place on patients and expressed dismay that some patients may die because they could not afford the full course of treatment. Recent negotiations have resulted in much lower DAA prices for Rwandans, but our results indicate that the financial burden experienced by patients will not be fully alleviated until the costs for HCV RNA testing and transportation are also addressed.

Several system-level challenges to care were also identified through the patient and healthcare provider interviews. Patients expressed frustration in the complicated nature of the care pathway, which required visits to separate health facilities for physician consultations, HCV RNA testing, and medication pick up. Similarly, difficulties in accessing HCV RNA testing and recording HCV RNA test results emerged through conversations with healthcare providers as a substantial barrier. Of particular note was patients’ responsibility for retrieving their HCV RNA results from the testing laboratory and then having to personally deliver them to the physician to be read and recorded. Our previous quantitative investigation indicated that nearly one in three patients were missing the HCV RNA result necessary to determine if SVR had been achieved [14]. Findings from the current qualitative investigation point towards difficulties in navigating and completing the full care pathway, including HCV RNA testing, as a substantial system-level barrier encountered by patients.

Additional system-level barriers included high workloads of healthcare providers and a lack of specialized training. Time pressures on HCV care providers have been cited previously as a challenge that can negatively influence both communication with patients as well as the quality of care patients receive [39]. The dedication of healthcare providers to become better at caring for patients with HCV was exemplified by their willingness to seek out educational materials on their own. Similar system-level barriers in the US have been moderated through implementing technology-centered solutions such as telemedicine [40] and on-line trainings. Current efforts to ameliorate these issues in Rwanda focus on more traditional decentralization and task shifting models, yet the institutionalization of internet-based reporting at lower-level health facilities suggests that technology-based interventions could be developed in the near future.

From the perspective of patients and healthcare providers, a number of interconnected enabling factors helped patients overcome the aforementioned barriers. Perhaps the most prominent of these facilitators was the positive relationship patients shared with the HCV healthcare providers. Patients expressed that physicians and nurses gave them hope for overcoming HCV and also provided comfort when patients were anxious about being diagnosed with HCV. This high degree of positivity reported by patients likely helped them see beyond the inconveniences caused by delayed appointments or short consultations to help them remain engaged in care. Supportive patient-provider relationships have been described as key facilitators to care for patients undergoing HCV treatment elsewhere [31, 35, 38] and it is promising that HCV providers in Rwanda are motivated as professionals and that patients share a fulfilling relationship with them.

The lack of stigma around HCV in Rwanda has likely contributed to the large degree of family and community support reported by patients. Patients received financial and emotional support from family and friends that, among other things, assisted them with transportation costs and accommodation while traveling to receive HCV services. The absence of self or social stigma facilitated patients remaining engaged in care and connected to their communities. Studies conducted outside of Rwanda have routinely identified stigma as a barrier encountered by patients diagnosed with HCV. This stigma is at least partially due to the widespread association of HCV with injection drug use and weakens patients’ motivation to engage with formal and informal support structures [41, 42]. On the contrary, the nearly non-existent level of HCV-related stigma in Rwanda is associated with a high degree of social support for HCV patients. Emerging from the interviews conducted with healthcare providers was the informal peer support groups that formed as patients attended appointments with their treating physicians. More formalized groups have now formed, such as the Rwanda Organization for Fighting Against Hepatitis; this peer-to-peer support network could continue to be strengthened as patients who have completed DAA treatment begin to provide emotional support and assistance to new patients navigating the steps involved in HCV treatment.

Lastly, the mild side effect profile of DAAs and the propensity of the first cohort of Rwandan patients to follow their treatment plan helped patients remain engaged in care. DAAs represent a new generation of drugs that have revolutionized HCV treatment, not only because of their high efficacy rates, but also because they are much more tolerable than previous treatment options. Physicians in Rwanda had previously only been able to prescribe interferon-based drugs, which are known for myriad side effects that result from near daily injections. When DAAs were initially launched in Rwanda, many of the first cohort of patients had likely been waiting for these new treatments to become available rather than beginning interferon-based treatment. These mild side effects, combined with the fact that the initial cohort of patients was made up of mostly older adults without a history of drug abuse, may have resulted in high adherence rates compared to those measured for interferon-based treatments [12].

Several limitations to this study warrant mention. Given that this study was conducted with the first cohort of patients who underwent DAA therapy, the findings would require confirmation for patients who are now being treated at district hospitals and by general practitioners rather than HCV specialists. Additionally, the nurses who participated in the study had notably limited HCV-specific training, with several mentioning that they had not received any specialized training for patients with HCV. Future studies could investigate the impact of training on healthcare providers’ perceptions of HCV care in Rwanda. This qualitative study did not intend to quantify the influence of specific factors on patients’ clinical outcomes. These results are the perspectives of patients and healthcare providers and can be used to inform follow-up investigations on areas in need of further development throughout the care cascade. Additional studies are needed into the rates of screening, treatment initiation, treatment completion, and long-term outcomes including cirrhosis and liver cancer as well as assessments of self-rated quality of life.

## Conclusions

To our knowledge, this is the first study to identify barriers and facilitators to HCV care from the perspectives of patients and healthcare providers in sub-Saharan Africa during the initial rollout of DAAs. We found a suite of interrelated individual- and system-level factors that enabled and impeded care. There was a high degree of consistency between patients and healthcare providers, suggesting that these factors are widespread in Rwanda. Results from this study can be used to design and enact evidence-informed interventions to help maximize the impact of DAAs as Rwanda moves towards HCV elimination.

## Data Availability

The datasets generated and analyzed during the current study are not publicly available since consent from participants to do so was not granted and the confidentiality of the participants cannot be guaranteed if the datasets were made public. Due to small number of individuals interviewed and the potentially identifiable information contained in the data, transcripts are not being made available to other researchers at this time.
